# Does the road to primary prevention of inflammatory bowel disease start from childhood?

**DOI:** 10.1002/jgh3.12782

**Published:** 2022-06-22

**Authors:** Saurabh Kedia, Vineet Ahuja

**Affiliations:** ^1^ Department of Gastroenterology All India Institute of Medical Sciences New Delhi India

There is always one moment in childhood when the door opens and lets the future in

‐Graham Greene

The search for nemesis of inflammatory bowel disease (IBD) has intensified in the recent decade and the immunological hubs are being throttled at multiple sites with the fond optimism that one of these countless hubs may be the holy grail. Despite this, the desultory of the disease is such that the treating physician would want to strike at the primary preventive level. Numerous studies and meta‐analysis have explored the possible factors that may trigger disease causation.^1^, ^2^, ^3^ The protective umbrella is comprised of factors like vaginal delivery, breastfeeding, absence of antibiotic use, diet consisting of fruits and vegetables, and childhood pets, and clearly the common vector out here is that right from birth time each of these factors assume a very vital role. And these childhood influences are reflective of future health. There has been lot of literature on how later life language skills and memory skills are dependent on early childhood training. And surprisingly, this appears to be very true for primary prevention of IBD too, as very early environmental influences decide the shape of things to come.

The epidemiology of IBD in developed and developing nations is expected to course through various stages of evolution as proposed by Kaplan *et al*.—the stage of compounding prevalence in developed nations, and the stage of emergence or accelerating incidence in the developing world.^4^ Industrial revolution, urbanization, improved sanitation, and dietary and lifestyle changes are considered responsible for this epidemiologic shift as the newly industrialized or developed societies mirror the IBD trajectory seen in the developed world,^5^, ^6^ though with a time lag of 3–4 decades.^7^, ^8^ This is reflected in the mounting prevalence of IBD in the two most developed countries of Asia—Japan and South Korea—where IBD prevalence increased from 76 and 24 per 100 000 in 2005 to 229 and 93 per 100 000, respectively in 2014–2015,^9^, ^10^, ^11^, ^12^ and the other Asian countries are expected to match this trend over the next three decades.^4^ With 60% of global population, the projected absolute IBD numbers in Asia may be equal to the West and are expected to surpass the Western figures,^13^ putting tremendous pressures on the already constrained healthcare resources in these regions. Hence, there is an urgent need to modify the pace of this epidemiologic evolution, through identification and mitigation of the environmental influences, which begin prenatally and continue to impact the IBD risk in perinatal period, infancy, childhood, and adolescence. With the genetic contribution being less than 30%,^14^ the environment contributes maximally to the IBD risk through its influence on the gut microbiome, epithelial barrier, and the local immunological milleu.^15^

Considerable efforts have been made globally in this direction, although there have been very few consistent and many more heterogeneous associations, which till date have precluded the development of any definite recommendation for prevention of IBD in high‐risk populations.^16^ Mak *et al*. have made a welcome addition to the existing, and should be congratulated for their efforts.^17^ The ENIGMA (Eastern Inflammatory Bowel Disease Gut Microbiota), a case–control observational study, evaluated the environmental factors in four different cohorts: three Chinese economies—Hong Kong (developed economy China), Guangzhou (urban China), Kunming (rural China), and Australia, and compared these risk factors between patients with Crohn's disease (CD), their first degree relatives (FDRs), household members (HMs), and unrelated healthy controls (HCs), through a linguistically and culturally validated ENIGMA Environmental questionnaire (EEQ). The study was unique in terms of including a genetically (FDRs) and environmentally (HMs) comparable cohort, in addition to unrelated HCs, which powered the study to dissect the environmental influences in the background of (epi)genetic similarity. Two contrasting observations (unlike previous findings) emerged from the study: positive influence of antibiotics on the risk of CD in Chinese population (in comparison to FDRs and HMs but not HCs), with the absence of any influence in Caucasians, and the absence of family history as a risk factor in the Chinese but not the Australian cohort. The direction and effect size of other factors were also heterogeneous in comparison to other global and Asian studies, and there was no association between breastfeeding and smoking. Although the antibiotics promoted the risk of CD in Asians, dose–response relationship could not be evaluated and discrepant association with respect to (epi)genetically similar and unrelated controls is intriguing. The possible explanation could be a tilt in the stability of already dysregulated microbiome in (epi)genetically predisposed individuals (FDs and HMs) under the pressure of antibiotics, which probably could not destabilize the more resilient microbiome in HCs. However, a recent large population‐based prospective case–control study from Sweden demonstrated a dose–response relationship between number of antibiotic courses (with a strong association for broad spectrum antibiotics) and IBD risk, which, unlike ENIGMA study, was consistent with both unrelated HCs and IBD‐free siblings.^18^, ^19^

Previous studies have identified an East–West divide in the association between antibiotic use and the risk of IBD, with a positive association in the West and negative association in studies from the East. In a previous prospective inception cohort study from the same authors, antibiotic use in childhood, breastfeeding, and pet exposure in childhood protected against CD in Asians, in comparison to asymptomatic controls (invited from streets and departmental stores in the same residential area).^20^ A recent meta‐analysis that compared environmental risk factors between West and East also echoed similar findings. Antibiotic use in childhood elevated the risk of CD in West, but reduced it in studies from East.^1^ In contrast to studies that have evaluated and compared the relationship between antibiotics and CD risk in native populations, an immigrant study assessed the CD risk in antibiotic‐exposed native Australians and Middle East immigrants in comparison to their respective HCs and reported similar associations.^21^ The possible reasons for the divergent association between antibiotic use and risk of CD in East and West could be related to antibiotic prescribing patterns and indications, differences in the method of data collection (registry based *vs* questionnaire), recall bias, sample size of included studies, and the possibility of antibiotic use being a surrogate of recurrent childhood infections, which in consonance with the hygiene hypothesis^22^ could have reset the microbiome and gut environment toward an anti‐inflammatory phenotype. However, a recent administrative database analysis from Japan demonstrated findings similar to the present study, and reported a positive association between antibiotic use (1 year before diagnosis) and CD risk.^23^ Does it mean that the direction of association depends upon the environmental factors such as prevalent hygiene and infection risk? In the developing countries where the risk of childhood infection is still high, antibiotics demonstrate a protective effect, and with improving sanitation and reduced childhood infections, the direction of effect reverses. The ACCESS cohort, which was analyzed almost a decade back, demonstrated a protective effect, while ENIGMA cohort demonstrates a positive association, possibly due to improvement in environmental conditions over the last decade. Similarly, Japan, which is the most developed economy in Asia, also showed a positive association between antibiotics and CD risk. This indicates that more important than genetics are the environmental or epigenetic factors that influence each other in determining the risk of IBD.

Like antibiotics, there are other factors that have a divergent association with CD risk in East and West, as demonstrated in the Asian prospective cohort study, Middle East migration study, the meta‐analysis comparing East and West, and a recent umbrella review of meta‐analyses^1^, ^2^, ^20^, ^21^ (Fig. 1). Smoking has consistently demonstrated a positive association with CD risk in West but heterogeneously so in East, with maximum studies demonstrating no association. Breastfeeding, though, is protective in both populations, the effect size is larger in Asians, and the factors related to hygiene (exposure to pets, bedroom sharing, number of household members), though heterogeneous, have demonstrated a stronger protective effect in Asians.^1^, ^2^, ^21^ Other than the divergence between populations (West *vs* East), discrepant effect has also been demonstrated within populations with respect to most of the early life risk factors. In a recent meta‐analysis of early life exposure and risk of IBD, the only protective factor was breastfeeding, and the harmful factors were passive smoking during antenatal period and otitis media in early childhood. Antibiotic exposure had a trend toward positive association and other factors such as mode of delivery, socioeconomic factors, hygiene‐related factors, child health, physical activity, and vaccination had uncertain significance.^3^

**Figure 1:**
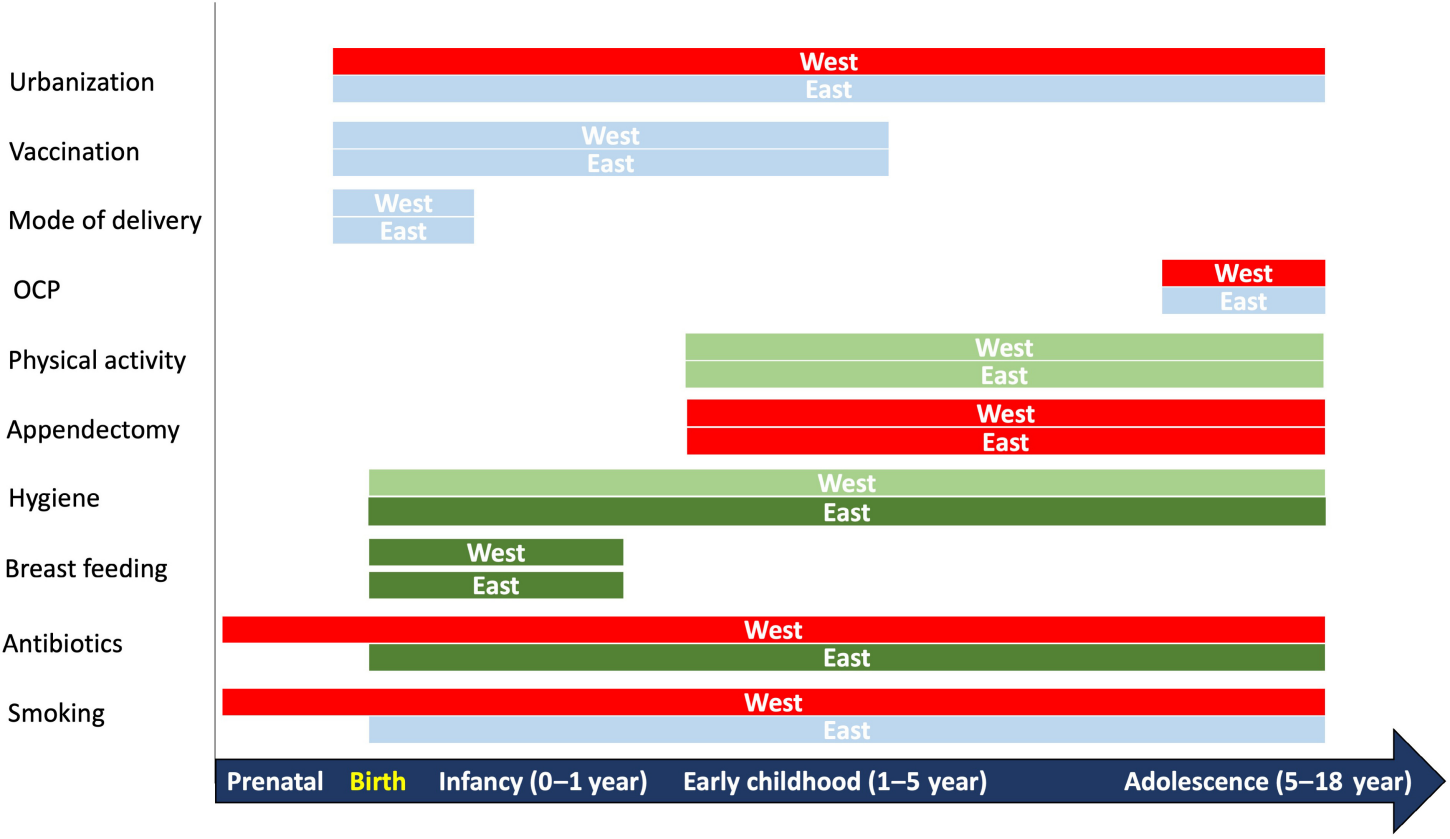
Comparison of association between environmental risk factors and risk of Crohn's disease in East and West. The bars in green indicate a protective effect, those in red indicate a positive association, and those in blue indicate no association. The intensity of color indicates the strength of association. The distance from the vertical axis represents the association of factors at various time points. These inferences have been derived from recent meta‐analyses.^1^, ^2^, ^3^ OCP, oral contraceptive pill.

The lack of family history as a risk factor for IBD in Asians, although was surprising, was in agreement with a low family history of IBD as reported in cohort studies from Asia.^24^, ^25^, ^26^, ^27^ The familial association of IBD could be due to shared genetic as well as epigenetic (same environmental influence) factors. Well‐described genetic loci such as NOD2, ATG16L1, and IRGM, which are involved in innate immune response against microbes have been associated with IBD from West but not in studies from Asia.^8^, ^28^ However, the similar or elevated IBD risk in South Asian immigrants in United Kingdom and Canada suggests a similar genetic predisposition to IBD in Asians,^29^ and this absence of reported genetic loci could be due to lesser number of studies conducted in these regions.

So, what do we infer from these observations? Does the present evidence assist us to formulate preventive strategies against the accelerating incidence/compounding prevalence of IBD? As with the heterogeneity associated with IBD phenotype, natural history, disease course, and treatment response, there is substantial heterogeneity in the observed associations between environment and IBD. The possible reasons could be biological or methodological. Biological differences could arise due to inherent differences between the populations assessed—East *versus* West, urban *versus* rural, children *versus* adults, migrant *versus* native, Caucasians *versus* non‐Caucasians, and due to differences in the included controls‐related *versus* unrelated, healthy *versus* diseased, same, or different geographical area, matched *versus* unmatched. Methodological discrepancies could be due to differences in study design (cohort *vs* case–control), timing of assessment (pre or perinatal, infancy, early childhood, adolescence), unmeasured confounders that arise due to lack of randomization at baseline, issue with reverse causality (when the risk factors are measured close to disease onset), and the method of measurement. Environmental risk factors or the exposome are difficult to measure or quantify, because of recall bias, missing data, interviewer bias, and lack of uniform definitions. Dose–response relationship if present (like with breastfeeding or antibiotic) could add strength to the association, in the absence of which the association remains plausible.

We again commend the authors for their well‐conducted study, which adds to the existing evidence and raises further questions related to this exciting and intriguing concept. However, we are still very distant from the conclusion and need global multicenter efforts with large, well‐designed, prospective cohort studies, which take care of the possible biological and methodological biases that have been identified within the current evidence. Undoubtedly, one would agree with Fredrick Douglass that “It is easier to build stronger children rather than repair broken men.”

## Funding support

Indian Council of Medial Research: Center for Advanced Research and Excellence in intestinal diseases 55/4/11/CARE‐ID/2018‐NCD‐II
